# Digital storytelling as a memory-making intervention for children and families in paediatric palliative care in Ireland: an adaptation study

**DOI:** 10.3389/fpubh.2025.1690798

**Published:** 2026-01-13

**Authors:** Razieh Safarifard, Aima Molati, Yvonne Corcoran, Gemma Kiernan, Eileen Courtney, John Mitchell, Terrah Foster Akard, Graham Moore, Veronica Lambert

**Affiliations:** 1 School of Nursing, Psychotherapy and Community Health, Dublin City University, Dublin, Glasnevin, Ireland; 2 Barretstown Children’s Charity, Barretstown, Ballymore Eustace, Co. Kildare, Ireland; 3 Vanderbilt University School of Nursing, Nashville, TN, United States; 4 Centre for Development, Evaluation, Complexity and Implementation in Public Health Improvement (DECIPHer), School of Social Sciences, Cardiff University, Cardiff, United Kingdom

**Keywords:** adaptation, digital storytelling, intervention, legacy building, memory making, paediatric palliative care

## Abstract

Memory making is a core component of holistic paediatric palliative care. However, traditional artifact-based keepsakes (e.g., handprints) are often passive and lack the developmental and cultural sensitivity needed for meaningful engagement. A more participatory, narrative-based, multimedia approach, such as digital storytelling, is therefore required. Following the ADAPT framework (Steps 1–2: Intervention-Context Fit and Planning Adaptations), this study adapted a U.S. developed digital storytelling intervention for children with life-limiting and life-threatening conditions and their families in Ireland. Methods included a literature review, stakeholder consultations (*n* = 21), co-production workshops, and the development of a facilitator training programme and delivery manual. The adaptation team comprised diverse stakeholders, including paediatric palliative care clinicians, creative practitioners, bereaved parents, and representatives from national children’s palliative care organisations. The adaptation, conducted in partnership with Barretstown Children’s Charity, yielded six key principles for the final intervention: emotionally safe framing, family and sibling inclusive design, flexible and multimodal participation methods, selective integration of therapeutic recreation, family-led pacing and facilitator preparedness. The final design incorporates play, visual, and audio elements to support meaningful, co-created engagement from all family members. Comprehensive training materials and a facilitator manual were developed to ensure fidelity and safety. This adaptation study presents a culturally and developmentally resonant digital storytelling intervention for Irish paediatric palliative care. Findings highlight the critical role of co-production, cultural fit, and emotional flexibility in successfully implementing complex psychosocial interventions for this population. Future pilot testing will evaluate the intervention’s feasibility, acceptability, and psychosocial impact.

## 1 Introduction

The growing global prevalence of children with life-limiting and life-threatening conditions (LLTCs) has increased the demand for paediatric palliative care (PPC). PPC aims to holistically address the physical, developmental, psychosocial, and spiritual needs of children and their families (1–3).

While the importance of PPC is recognised globally, substantial service gaps persist, particularly in the provision of accessible psychosocial support and structured home-based care (4–6). To bridge this gap, psychosocial interventions focused on legacy-building and memory-making have emerged as vital components of holistic PPC. This paper details the structured, multi-phase process used to adapt an evidence-based digital storytelling intervention to align with the specific cultural and clinical context of PPC in Ireland.

### 1.1 Background and context

#### 1.1.1 The need for structured psychosocial support

In Ireland, service demand for PPC has grown alongside global trends (7, 8). However, service provision is often fragmented, resulting in persistent gaps in family support and access to home care (8, 57). This lack of structured support can place significant burdens on families and healthcare providers. As noted by Timóteo et al. (9), nurses and other healthcare providers need structured, evidence-based tools to effectively meet the complex needs of these children and families.

#### 1.1.2 Legacy-building interventions

Legacy-building is an increasingly important component of PPC understood as a dynamic, relational process of co-creation of meaning and memories (10, 11). Its primary goals are to document meaningful legacies, build emotional resilience, and strengthen family bonds (12–14). A range of legacy-making interventions are used in PPC, particularly in the terminal phase, including hand and footprint moulds, personalised artwork, memory books, photographs, video recordings, and participatory storytelling (12–15, 16, 60). These approaches vary in complexity and emotional intensity and are often selected based on the child’s developmental stage, communication ability, and energy levels. For instance, children with limited verbal capacity or fatigue near end-of-life may benefit from low-burden, proxy-supported interventions such as therapeutic videography or collaborative art-making (15, 60), while older children and adolescents may prefer narrative-led, autonomy-supportive models such as digital storytelling or dignity therapy (12, 14, 17). These methods support emotional expression, identity preservation, and connection with family members.

While traditional, low-burden keepsakes (e.g., handprints, photographs) offer tangible comfort to grieving parents (18–20), participatory memory-making gives children greater voice and agency in shaping their own legacies (15). Recent concept analyses highlight that the value extends beyond the creation of the object itself to the relational and ritualised process of co-creation, which fosters connection and continuity (9, 21).

#### 1.1.3 Dignity therapy and digital storytelling

Structured interventions, specifically dignity therapy and digital storytelling, enhance this engagement by helping children reflect on their values and experiences in developmentally appropriate, culturally sensitive formats (17, 22–25).

Dignity Therapy, originally developed for adults in palliative care, provides a structured way to reflect on personal values, meaningful experiences, and legacy (24, 26). While its effectiveness in reducing distress and enhancing meaning at the end of life is well established in adults (25), its direct use with children is limited (27). Recent adaptations, however, have incorporated creative elements such as art, video, photography, and storytelling, making dignity therapy more engaging for children, enabling them or their proxies to co-create meaningful legacies that support memory preservation and emotional expression (27, 28).

Digital storytelling is an evolution of these creative approaches, building on the same principles by incorporating multimedia elements (video, audio, photographs) to create interactive, evolving narrative format, distinguishing it from static, artifact-based memory-making (22, 23, 29–32). Research indicates that storytelling enhances emotional expression, strengthens parent–child communication, and promotes adaptive coping (33–35). Studies have shown the feasibility and positive outcomes of digital storytelling among paediatric cancer patients, including enhanced family connections and reduced emotional distress (23, 29, 36, 37, 61).

#### 1.1.4 The critical need for adaptation

Effective implementation of these interventions requires rigorous cultural and developmental adaptation. Current models often focus on adolescents and lack structured, family-centred formats for diverse PPC populations (31, 37, 38, 58). For example, while cultural adaptations have occurred in places like China (17), Portugal (25) and the United Kingdom (39), no structured evidence-based digital storytelling protocol has been formally adapted for the Irish PPC context where storytelling holds deep cultural significance (19, 40). The efficacy and safety of these protocols are contingent on rigorous adaptation to the local context.

### 1.2 Purpose and contribution

#### 1.2.1 Specific research questions

The systematic adaptation process was guided by the following research questions:

1. What are the cultural, developmental, and contextual factors that influence the successful implementation of a digital storytelling intervention in the Irish PPC setting?
2. How can an existing evidence-based digital storytelling protocol be systematically adapted to enhance its cultural resonance, developmental appropriateness, and emotional safety for Irish children with LLTCs and their families?

#### 1.2.2 Study purpose and contribution

The purpose of this study was to systematically adapt an existing digital storytelling memory-making intervention, originally developed in the U.S. for children with advanced cancer, for use with Irish children with LLTCs and their families. Conducted in partnership with Barretstown Children’s Charity, the process adhered to a formal adaptation framework.

The contribution of this study is two-fold: first, it provides the systematic adaptation of an evidence-based legacy-building protocol to the Irish PPC context, providing a rigorous methodological framework for similar future translations, and second, it details the six guiding adaptation principles (emotionally safe framing, family and sibling inclusive design, flexible participation methods, selective therapeutic recreation integration, family-led pacing, and facilitator preparedness) that resulted from this process. These principles are integral to the resulting culturally resonant facilitator manual, which supports consistent and safe delivery in Ireland. Adhering to a formal adaptation process, this study contributes a transparent, reproducible framework for tailoring psychosocial interventions to new settings.

#### 1.2.3 Theoretical framework

The ADAPT Guidance for Adaptation of Interventions (41) served as the theoretical framework for this adaptation study. This model offers a structured, multi-phase process to systematically adapt evidence-informed interventions to new settings. Our adaptation aimed to enhance the developmental appropriateness, cultural resonance, and emotional safety of memory-making interventions for Irish children with LLTCs and their families. This theoretical grounding guided the overall strategy, including the steps of contextual analysis, stakeholder engagement, and the co-development of a facilitator manual to support safe and consistent delivery. The resulting six guiding principles (detailed in the Discussion) represent the practical application of the ADAPT model, translating theoretical steps into concrete, context-specific intervention components.

## 2 Methods

This study followed the ADAPT guidance (41) to systematically adapt the original evidence-informed intervention to the Irish context. The ADAPT model outlines four interrelated steps, emphasising that systematic adaptation is crucial for achieving a good fit and enhancing effectiveness and implementation. This paper reports specifically on the activities conducted during ADAPT Steps 1 and 2. These steps were operationalised across four iterative phases, with stakeholder involvement integrated as an overarching principle throughout the entire process.

### 2.1 ADAPT step 1: assessing intervention–context fit

#### 2.1.1 Phase 1 – identifying and assessing an existing evidence-informed intervention

To identify a suitable evidence-informed intervention for adaptation, we conducted a systematic review of memory-making support for children with LLTCs and their families (13, 14). The review identified three categories of interventions: (1) storytelling-based, (2) art-based, and (3) physical keepsakes. Digital storytelling was identified as the most suitable intervention due to its strong empirical foundation. Multiple studies have demonstrated its feasibility and acceptability in paediatric oncology and palliative care, with potential benefits for emotional expression, family communication, and memory preservation (22, 23, 29–31, 36, 37, 61).

Following this selection, we assessed the intervention’s relevance and transferability by reviewing its core and adaptable components. Using the Template for Intervention Description and Replication (TIDieR) checklist (42), we analysed the fundamental aspects of Akard et al.’s work, which highlighted the intervention’s potential to enhance child-family bonds and provide emotional and psychological benefits. In parallel, we initiated informal consultations with PPC professionals and creative practitioners in Ireland to identify context-specific considerations, such as cultural values, existing service structures, and potential delivery settings. This aligns with the ADAPT framework’s emphasis on understanding the local context.

#### 2.1.2 Phase 2 – stakeholder consultations

##### 2.1.2.1 Stakeholder composition and selection criteria

Guided by the ADAPT framework (41), this iterative phase involved the intentional selection of stakeholders (*n* = 21) who possessed diverse and essential expertise for the adaptation process. The core adaptation team was chosen based on specific criteria designed to ensure local relevance, cultural resonance, and implementation feasibility:

- Professionals specialised in PPC, bereavement support, and national advocacy (e.g., representatives from LauraLynn Children’s Hospice, Irish Cancer Society, Jack and Jill Children’s Foundation, and the Irish Hospice Foundation).
- Practitioners with experience in therapeutic recreation and service delivery from Barretstown Children’s Charity (the primary co-production/knowledge user partner).
- Bereaved parents who participated as active collaborators, providing crucial public and patient involvement (PPI) insight into emotional safety and cultural fit.
- Academic researchers and specialists familiar with the original digital storytelling protocol.

Stakeholders participated in an advisory and co-productive capacity, rather than as participants for qualitative data collection.

##### 2.1.2.2 Adaptation process and documentation

The multi-stakeholder consultations focused on assessing the Intervention-Context Fit and Planning Adaptations (ADAPT Steps 1 and 2). Input was structured across five key domains: identifying adaptation needs, assessing contextual fit, co-designing the intervention, planning facilitator training, and establishing feasibility and acceptability metrics. The outcomes from these sessions informed iterative modifications to the intervention’s language, format, and facilitation roles. Key insights and decisions were captured in a bespoke iterative adaptation matrix, which is grounded in the ADAPT framework. This matrix served as a systematic tool for documentation and decision-making, not formal qualitative analysis, by mapping stakeholder consensus and translating input into concrete actions (retention, modification, or reframing of specific intervention components). To support accurate recall and summary of these decision-making processes, all consultation sessions were audio-recorded (used for detailed field notes and decision verification, not research analysis) and supplemented by detailed field notes. No identifiable data were retained. Formal ethical approval was not required as stakeholders participated in an advisory and co-production capacity and were not considered participants or data providers.

### 2.2 ADAPT step 2: planning and undertaking adaptations

#### 2.2.1 Phase 3 – co-production and adaptation of intervention content

Consistent with the ADAPT framework, we held four co-production workshops with Barretstown’s team members to guide the adaptation of the intervention’s core elements. During these sessions, the team collaboratively co-developed the intervention’s structure, session flow, and guiding questions for storytelling, seamlessly integrating Barretstown’s unique therapeutic recreation model and multimedia elements. Key adaptations included reframing the intervention to be more family-centred by integrating sibling participation and developing flexible tools for children with limited verbal ability or fatigue.

Further insights from the workshops were captured through facilitated reflection and documented in the adaptation matrix. This process made transparent how collaborator input translated into concrete changes, yielding six guiding adaptation principles. All adaptations were systematically documented using the TIDieR checklist to clarify what was retained, reframed, or newly introduced for the Irish context. These documented outputs then informed the development of training and implementation materials, with the full chain of decisions summarised in a companion adaptation decision log. Throughout this process, collaborators continued as co-producers rather than research participants, with all engagement remaining advisory and no identifiable data were retained.

#### 2.2.2 Phase 4 – developing training and implementation materials

This phase focused on developing robust training and implementation materials to ensure the adapted intervention could be delivered effectively in practice, a key tenet of the ADAPT framework. While the original U.S. studies delivered the intervention with a paediatric nurse specialist (22) or through a web-based, family-led format (29, 31, 36, 37, 61), our Irish adaptation required a workforce adaptation. Delivery will be primarily by Barretstown’s Outreach Team—therapeutic recreation specialists with expertise in family engagement and bereavement-informed practice.

**Figure 1:**
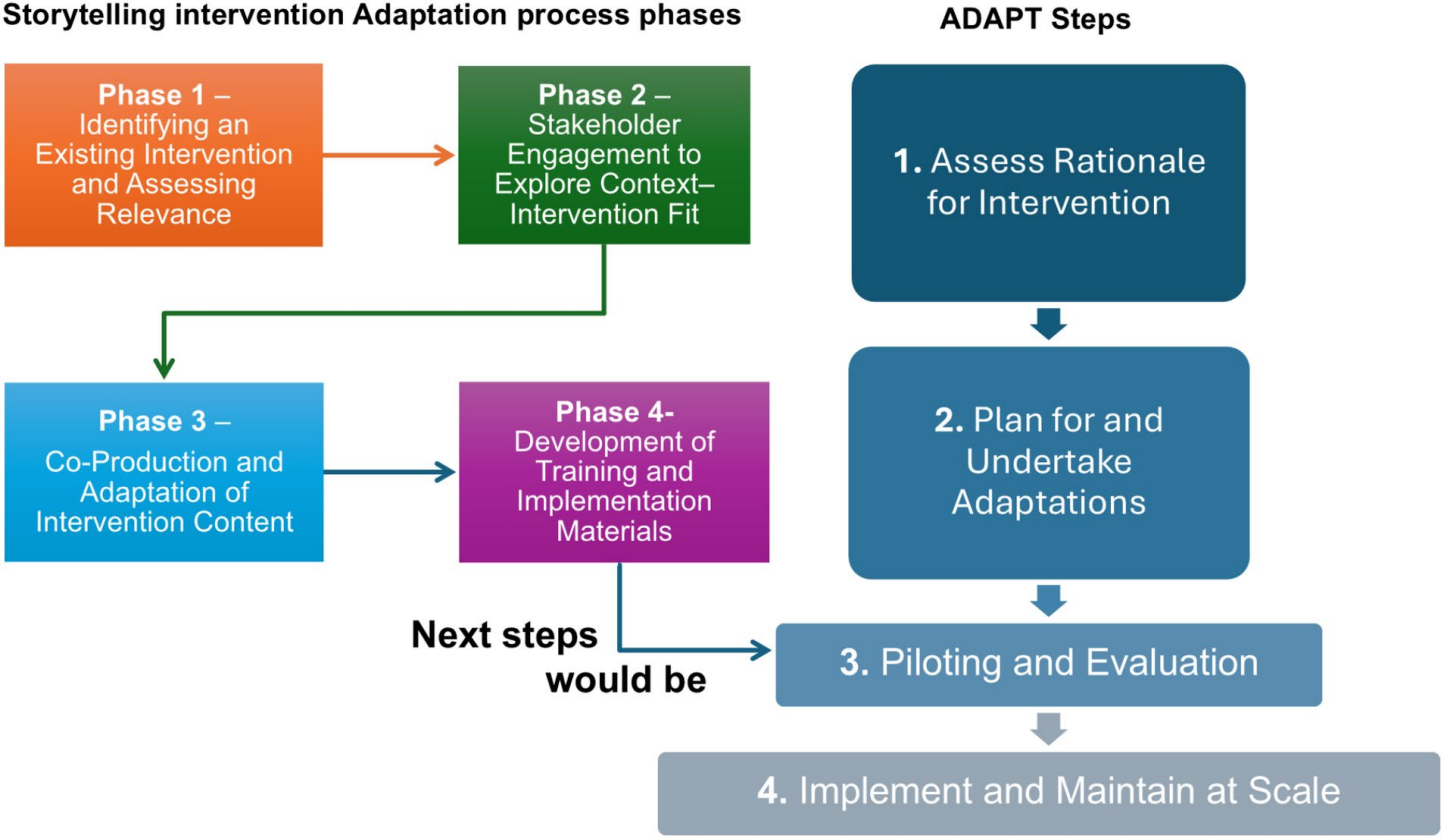
The ADAPT steps and the phases used to adapt the digital storytelling intervention.

A comprehensive facilitator training programme was developed and delivered across four online sessions. The training covered key topics such as bereavement-informed communication, building emotional resilience, and practical digital storytelling techniques, with a focus on a family-centred delivery approach. Role-play and supervised practice sessions were incorporated to ensure facilitators were confident in delivering the adapted intervention in home-based settings, maximising its therapeutic potential and fidelity to the adapted model. We finalised the intervention manual alongside the training, integrating all adaptations and providing detailed session guidance and safety protocols. Field notes and reflective observations from training sessions were used to inform final adjustments to the manual, ensuring clarity and feasibility before its use in a small-scale pilot phase (see Figure 1).

## 3 Results

This section presents the findings from the first two steps of the ADAPT framework (assessing rationale and planning adaptations). We have structured our results in line with the four-phase adaptation process: Phase 1 – identifying and assessing an intervention; Phase 2 – engaging stakeholders; Phase 3 – co-producing the adapted intervention content; and Phase 4 – developing training and implementation materials.

### 3.1 Phase 1 – identifying and assessing an intervention

Our systematic literature review highlighted a critical unmet need in PPC for memory-making interventions that are both developmentally appropriate and culturally responsive (13, 14). While current practices often rely on physical keepsakes like handprints, footprints, and photographs (19), these frequently lack the personalisation and meaningful child involvement needed to truly support emotional processing and family communication.

This review (14) demonstrated that the digital storytelling memory-making intervention offers a transformative, narrative-based alternative. It not only yields tangible legacy artefacts but also showed feasibility and acceptability in several pilot studies in the United States. Families reported improvements in emotional expression, parent–child communication, parent–child bonds and parent coping strategies (22, 23, 29–31, 36, 37, 43, 61). Although the effectiveness has not yet been confirmed by large-scale randomised controlled trials, children reported non-significant improvements in procedural anxiety (Cohen’s *d* = 0.35) and perceived physical appearance (Cohen’s *d* = 0.28) compared to a wait-list control group (37). The evidence supported its co-productive, narrative-based mechanism and its flexibility for local adaptation (30, 31, 36, 37, 61), confirming the intervention’s potential.

### 3.2 Phase 2 – engaging stakeholder and planning adaptations

Stakeholder consultations were conducted to translate the evidence base into contextually relevant design priorities for Irish PPC. By mapping the original intervention and Barretstown’s therapeutic recreation model using the TIDieR checklist (see Supplementary Table 1, a TIDieR summary is presented in Table 1), we identified a strong functional fit. However, consultations also highlighted the need for adaptations, including increased cultural sensitivity in language, broader participation for all family members, and greater flexibility for children with low energy or communication challenges.

**Table 1:** Overview of core and adaptable features of the digital storytelling intervention.

TIDieR dimension	Description (core components)	Adaptation features
WHY	Support memory making and emotional expression for children with LLTCs and their families, while strengthening family connection and shared memories.	Not applicable (core purpose retained).
WHAT	Creative storytelling tools and prompts (drawing, music, voice, photographs), co-produced digital stories reflecting each child’s preferences and strengths.	Language and framing: adjusted to reflect Irish cultural sensitivities and identity-focused storytelling.
WHO	Delivered by trained Barretstown Outreach Team facilitators. Supported by academic researchers and PPC specialists.	Delivery workforce: Adapting the original U.S. model (paediatric nurse specialist) to Barretstown’s therapeutic recreation specialists. Training: Enhanced to include grief literacy and emotional resilience in addition to technical training.
HOW	Flexible storytelling sessions using multimodal methods (drawing, audio, video) tailored to the child’s needs, communication styles and energy levels; with optional family or sibling involvement.	Participation: All family members and siblings can be involved. Format: Multimodal tools (drawing, music, audio, photographs) tailored to each child.
WHEN/WHERE	Delivered in-home via Barretstown trained staff. Sessions are 1–2 h and flexibly scheduled to align with each family’s emotional readiness and availability.	Delivery: Home-based sessions with flexible timing and pacing of families.

### 3.3 Phase 3 – co-production and adaptation of intervention content

Building on insights from consultations, co-production workshops with Barretstown’s team refined the intervention’s structure, session flow, and storytelling prompts. The selective integration of Barretstown’s therapeutic recreation approach allowed for the creation of engaging, home-based sessions without imposing the full structured camp model.

The iterative adaptation matrix captured the progression from stakeholder insight to concrete adaptation decisions throughout this phase. This process led to the development of six guiding principles that shaped the adapted intervention’s language, content, delivery, and implementation support. These principles ensured the final digital storytelling intervention was sensitive, empowering, and family-centred. The logic connecting consultation insights to the final adaptation decisions is summarised in Table 2.

**Table 2:** Adaptation decision log linking stakeholder insights to intervention changes.

ID	Principle	Stakeholder insight	Adaptation decision	Implementation in the Irish context
1	Emotionally safe framing	Language implying “goodbye” caused distress for families	Reframed intervention as life-affirming storytelling	Sessions celebrate identity; prompts avoid end-of-life framing
2	Family and sibling inclusivity	Families wanted siblings and parents actively involved, especially in home settings	Expanded sessions to actively facilitate joint reflection and expression across the entire family unit	Manual includes sibling and family-friendly tools like drawing, music, allowing families to co-create stories together
3	Flexible, personalised participation	Children with low energy or limited communication need alternatives	Added emotion cards, visual prompts, drawing tasks, and audio/video options	Children and their families choose multiple expression tools and modes
4	Selective therapeutic recreation	Full Barretstown recreation cycle too structured for home-based PPC	Integrated only playful, creative elements of the model	Focused fun-centric practices maintain engagement without overstructure.
5	Family autonomy and pacing	Families wanted control over timing, story and final product especially during bereavement	Adopted family-led protocols allowing families to choose when and how the digital story is finalised	Families set the pacing and format preferences during a pre-intervention call
6	Facilitator preparedness	Facilitators may face emotional strain	Developed psychological preparation, resilience training, and ongoing support	Training covers emotional resilience, digital tools, ethical communication, and child engagement strategies and includes mock-up sessions

### 3.4 Phase 4 – developing training and implementation materials

A comprehensive facilitator training programme, delivered in four online sessions, was a key step in preparing the Barretstown team. Facilitators reported that the training increased their confidence and skills related to trauma-sensitive practice, emotional resilience, and participatory storytelling design. The practical components, including role-play and supervised practice, were particularly valuable. Feedback and reflective observations from these sessions informed final adjustments to the manual, ensuring the materials were clear, feasible, and aligned with facilitators’ needs.

The finalised intervention manual (Supplementary Table 2) serves as a core tool for implementation. It incorporates all the co-produced adaptations, providing facilitators with step-by-step guidance, optional prompts, and comprehensive safety protocols. This resource ensures that the intervention can be delivered consistently while remaining flexible enough to be culturally grounded and responsive within home-based palliative care settings. The manual and training programme together establish a clear, documented approach to implementation, which will be tested in the upcoming pilot phase.

## 4 Discussion

This study systematically adapted a U.S. -developed digital storytelling intervention for Irish PPC, aiming to create a culturally relevant, developmentally appropriate, and emotionally safe memory-making experience. Using the ADAPT framework, the process involved a literature review, stakeholder engagement, co-production workshops, and the development of tailored training. The application of this framework addressed both guiding research questions by identifying contextual needs and developing a systematic strategy to meet them.

The methodology employed in this systematic adaptation was abductive reasoning, commencing with a deductive foundation rooted in the structured application of the ADAPT framework (41) to fit an existing evidence-based intervention to the specific Irish PPC context. The contextual factors influencing implementation (Research Question 1) were identified through iterative stakeholder consultations (an inductive phase), highlighting the need for increased cultural sensitivity in language, broader participation for all family members, and flexibility for children with communication challenges. The resulting six guiding principles (Section 4.1) answer Research Question 2 by detailing how the existing digital storytelling protocol was systematically adapted to enhance its cultural resonance, developmental appropriateness, and emotional safety for Irish children with LLTCs and their families.

The decision to adapt digital storytelling for Irish PPC was driven by evidence highlighting its feasibility, emotional value, and potential for personalisation. Digital storytelling interventions, where families co-create multimedia narratives, have demonstrated benefits in promoting communication, reducing emotional stress, and supporting anticipatory grief (12, 14, 22). Recent reviews emphasised that digital legacy tools, including digital storytelling, can enhance family connection and psychosocial well-being when integrated within supportive and flexible contexts (12, 14, 57). However, while many interventions are feasible, their effectiveness is dependent on cultural and contextual alignment. This systematic adaptation affirms the critical importance of tailoring the digital storytelling intervention to respect cultural values, family dynamics, and preferred modes of storytelling.

### 4.1 Six guiding principles: synthesis of adaptation

The systematic adaptation process achieved its purpose by translating the goal of cultural fit into a set of six practice-oriented principles. These six guiding principles shaped the culturally sensitive modifications to the intervention’s content, delivery, and implementation strategy (see Table 1). We now explore these principles in detail, as they informed the creation of a refined, adapted digital storytelling manual for the Irish context.

#### 4.1.1 Cultural and emotional sensitivity in language and framing

A key finding was the need to frame the intervention around life, joy, and meaning-making, rather than closure or loss. This approach aligns with Irish cultural traditions, where storytelling is deeply rooted in family and community life, and with literature on paediatric memory-making that emphasises reinforcing a child’s identity over foreshadowing death (19, 38). To ensure developmental appropriateness and reduce emotional risk, we avoided abstract prompts, such as “If you could give your family anything.” While no negative reactions were reported in the original studies, children rarely chose to include these types of prompts in their stories (44), a finding consistent with other adaptation studies (17, 25, 27). This principle also guided the use of emotionally safe language, focusing on “life stories” instead of “end-of-life” narratives, which aligns with critiques that caution against intensifying grief through premature or emotionally charged language (18, 38).

#### 4.1.2 Family-centred and sibling-inclusive storytelling

The adapted intervention transitioned from the original parent–child dyad sessions to a fully family-centred and sibling-inclusive model. This modification not only aligns with Barretstown’s approach of working with the family as a unit but also facilitates joint reflection and expression across the entire family, which is crucial for processing anticipatory grief and co-constructing legacy (27, 29). This approach is consistent with a growing recognition that memory-making is a co-creative process that can strengthen parental coping mechanisms and enhance psychosocial benefit for all family members (17, 30, 31, 57, 58).

#### 4.1.3 Tailored participation methods and personalisation

Another key principle was the need for flexible and personalised storytelling to accommodate the diverse needs of children with LLLTs. Recognising variations in energy, verbal ability, and sensory preferences, the intervention integrated multiple formats like play, drawing, and audio recording. These personalised, child-led approaches are consistent with trauma-informed principles that prioritise safety and agency through the digital storytelling sessions (35, 45) and reflect a broader commitment to dignity-based care by giving families meaningful choices and shared decision-making (27).

#### 4.1.4 Selective integration of Barretstown’s therapeutic recreation model

The adaptation selectively integrated Barretstown’s core principles of fun, flexibility, and child-led engagement of “challenge by choice” into a home-based setting. This approach avoided imposing the full structured camp model, ensuring that the digital storytelling sessions felt empowering rather than burdensome. This selective integration mirrors other child-centred therapeutic models (46, 47, 59) and aligns with existing adaptation guidance that emphasises preserving core values while tailoring delivery to the local context (41).

#### 4.1.5 Respecting family autonomy and readiness

The intervention was designed to respect families’ emotional timing and their right to choose how and when to participate. This principle empowers families to pause, adjust, or delay their involvement based on their immediate emotional capacity, a provision that affirms their autonomy and supports emotional safety (27, 48, 49, 56). This high degree of flexibility, while crucial for family-centred care, may pose a challenge for future evaluation, as balancing this with the standardisation needed for efficacy testing will be a key consideration.

#### 4.1.6 Facilitator preparation and emotional resilience

The final principle addressed the need to prepare facilitators for the emotional demands of memory-making. Unlike the original U.S. model led by a paediatric nurse, our Irish adaptation is led by Barretstown’s therapeutic recreation specialists, necessitating a workforce adaptation. The intensive training programme – combining psychological preparation with participatory storytelling workshops – equipped facilitators with essential skills in trauma-sensitive practice and emotional resilience. This approach ensures the intervention can be implemented safely, consistently, and without undue emotional burden for either families or facilitators (25, 49, 50, 56).

While stakeholder feedback was highly supportive, we acknowledge that memory-making may not be suitable for all families. Research has shown that some families may choose not to engage with memory products or may experience distress during early bereavement (20, 51, 56). These findings highlight the importance of flexibility and the need for facilitators to be trained in emotional readiness and trauma-informed care (52, 53). In addition, digital divide and access barriers—such as variable home bandwidth, device availability, or technical literacy—may limit the intervention’s reach, particularly in remote or underserved communities (31). The time and training demands on staff to support and edit personalised stories also present scalability challenges that will be explored during the pilot phase. Future research will involve a pilot phase to test the feasibility and acceptability of this adapted intervention in practice and to further explore the balance between fidelity and flexibility.

### 4.2 Theoretical and methodological limitations

This study has several theoretical and methodological limitations that should be acknowledged.

First, the adaptation was conducted in close partnership with Barretstown Children’s Charity, which means the intervention design is inherently shaped by this organisational context. Theoretically, the study is limited by the challenge of cultural transferability when adapting an intervention from a specific US context to the cultural and healthcare system of Ireland. While we systematically addressed contextual fit through co-production, the underlying assumptions of the original protocol may still influence the adapted design. Consequently, although we sought national applicability, some components may require further refinement before being implemented in diverse PPC settings or alternative service models.

Second, while we engaged a diverse group of 21 adult stakeholders, including bereaved parents, the consensus views derived from this process may not represent the full spectrum of perspectives across Ireland. The absence of regional variation, differing service structures, and under-represented diagnoses limits the breadth of contextual insights captured. Crucially, children and adolescents with life-limiting conditions did not participate directly in this adaptation phase. Although adult stakeholders provided invaluable proxy perspectives, the lack of direct input from the target population limits the developmental specificity of some adaptation decisions. Their involvement will be essential during feasibility testing and subsequent refinement.

Third, the boundaries between ADAPT Step 1 (identifying needs) and Step 2 (planning adaptations) were fluid and non-linear in practice. Stakeholder consultations and co-production workshops often generated overlapping insights, with adaptation priorities and concrete changes emerging in parallel. While this iterative process reflects the reality of intervention adaptation, we have acknowledged this methodological overlap by combining our reporting around the key guiding principles.

Fourth, a key clinical limitation is that memory-making interventions may not be suitable for all children and families in palliative care. Research indicates that a family’s readiness to engage with legacy activities can vary, and for some, the process may cause distress or feel emotionally premature (48, 49). While our adaptation prioritised flexibility and family autonomy to mitigate this risk, our study did not include direct input from families who might choose not to participate in the intervention. This represents a limitation in understanding the perspectives of those who may not be ready or willing to participate in legacy-building, an important area for future research.

Finally, this manuscript focuses exclusively on the foundational adaptation process (ADAPT Steps 1 and 2). Methodologically, this work represents an intervention adaptation process only. Consequently, the findings cannot speak to the intervention’s real-world feasibility, acceptability, or effectiveness. These critical variables remain the subject of necessary future pilot testing.

## 5 Conclusion

This study adapted a U.S.-developed digital storytelling intervention for use within the Irish PPC context, employing a multi-phase process guided by the ADAPT framework. Our systematic approach, driven by stakeholder co-production, yielded a refined psychosocial intervention that champions developmental appropriateness, cultural resonance, and emotional safety. The fundamental insight of this work is that effective legacy-building is not about producing passive, artifact-based keepsakes; it is about transforming the process into a highly relational, meaning-making journey. The adaptation process achieved this by reframing the intervention around life-affirming storytelling and integrating playful, visual, and audio elements to facilitate family-centred and sibling inclusive engagement. The resulting six-guiding principles (e.g., emotionally safe framing, flexible participation, family-led pacing) provide a practical, culturally resonant blueprint for delivering complex psychosocial support in a specialised field. By systematically tailoring the core intervention to reflect the unique values and lived experiences of Irish families, the findings emphasise the role of cultural fit, co-production, and emotional flexibility in implementing psychosocial interventions for children with life-limiting conditions. This systematic work lays crucial groundwork for future implementation, with the next step being to pilot test the feasibility and acceptability of this refined digital storytelling approach within the Irish PPC setting.

### 5.1 Implications and future directions

This systematic adaptation study of a digital storytelling intervention offers valuable insights for psychosocial intervention work in PPC and outlines pragmatic pathways for future research and practice. This work demonstrates the necessity for culturally informed adaptation when integrating health interventions into new contexts. Drawing on structured frameworks like ADAPT, alongside stakeholder co-production, was instrumental in shaping an intervention that is not only contextually appropriate but also genuinely resonates with the specific values and lived experiences of families. This iterative and responsive approach, carefully documented through tools such as the TIDieR checklist, highlights that effective adaptation is rarely linear; it demands continuous dialogue, flexibility, and a genuine responsiveness to local insights.

#### 5.1.1 Implications for clinical practice

Beyond simply refining an existing tool, the principles derived from this process highlight several implications for clinical care:

- By empowering families to actively co-create their stories, we encourage a process of meaning-making that moves beyond passive memory preservation. These insights offer a practical blueprint for integrating narrative-based, participatory approaches that foster emotional safety and personal relevance during profoundly challenging times.
- The principles of emotionally safe framing and family-led pacing (as outlined in our results) should be adopted as standard practice for any legacy-building activity, ensuring that the intervention is tailored to a family’s readiness and autonomy, thereby mitigating the risk of emotional distress highlighted in our limitations section.
- The complex, sensitive nature of narrative work demands specialised training. Organisations should invest in comprehensive training and supervision models to ensure facilitators possess the necessary clinical, digital, and creative skills to maintain fidelity and safety.

#### 5.1.2 Implication for future research

Building on this foundational adaptation, the immediate next step is to systematically pilot the adapted digital storytelling intervention with families receiving PPC in Ireland.

- Acceptability: To understand family engagement levels, their emotional responses, and their perceived value of the final digital product in a real-world setting.
- Feasibility: To evaluate logistical demands, the training needs of facilitators, and the optimal timing and duration for intervention delivery within the realities of palliative care provision.
- Qualitative experiences: To gather data on families’ experiences of both the storytelling journey and the digital legacy created, emphasising its emotional resonance, personal significance, and perceived long-term impact on their grief and remembrance. Crucially, this phase will directly address the limitation of lacking child and adolescent input by systematically gathering their feedback.

Following initial piloting, future research should explore

- Investigating the scalability and broader applicability of this adapted intervention across various PPC settings, both nationally and internationally.
- Studies should investigate the longer-term psychosocial outcomes for families, including potential impacts on bereavement processes, family cohesion, and overall well-being.
- Research should explore workforce models, cost structures, integration into routine care, and long-term digital storage solutions that protect family ownership, privacy, and autonomy. As demonstrated by Cho et al. (31), home-based legacy interventions may be feasible and meaningful, though digital literacy, bandwidth access, and timing in the illness trajectory must be considered.

Additionally, there is growing potential to incorporate optional, ethically governed uses of emerging technologies. Recent scholarship suggests that artificial intelligence tools, such as adaptive story prompts, voice-to-text transcription, or emotion-aware scaffolding may support families in co-creating digital legacies, especially when communication or energy is limited (54, 55). These tools should never replace human facilitation or automate a child’s narrative. Rather, they may enhance accessibility and emotional support when designed with robust safeguards: explicit consent, on-device data processing, family control of outputs, and trauma-informed usage guidelines.

#### 5.1.3 Implications for policy and management

Ultimately, this work contributes to a realistic understanding of how compassionate, culturally attuned psychosocial interventions can be thoughtfully developed and integrated. Policy should support resources for systematic adaptation methodologies like ADAPT, ensuring that intervention development is seen as a necessary precursor to efficacy testing. Management teams in PPC should prioritise and resource implementation research to ensure successful integration into routine care, opening avenues for supporting children and families through some of life’s most challenging journeys.

## Data Availability

The original contributions presented in the study are included in the article/Supplementary material, further inquiries can be directed to the corresponding author.
