# Supplementary material for: Digital storytelling as a memory-making intervention for children and families in paediatric palliative care in Ireland: an adaptation study
Source: Front Public Health. 2026 Jan 13;13:1690798. doi: 10.3389/fpubh.2025.1690798 (PMC12835261; doi:10.3389/fpubh.2025.1690798)
Supplement: Supplementary file 1 [file Table_1.docx]

**Supplementary File 1 - Mapping the original intervention and adapted intervention using the TIDieR checklist**

| TIDieR Item | Description | 1.Legacy Building Intervention (USA) | 2. Digital storytelling intervention (Ireland) | Similarities | Differences | Note |
| --- | --- | --- | --- | --- | --- | --- |
| Brief Name | Provide the name or a phrase that describes the intervention. | Legacy Building / digital storytelling Intervention (USA) | Digital storytelling Intervention at Barretstown and Barretstown Home Camp | Both aim to support children with serious illnesses and their families. | Digital storytelling (Ireland) intervention includes a wider range of activities beyond digital storytelling. |  |
| Why | Describe any rationale, theory, or goal of the elements essential to the intervention. | Improve quality of life and communication, offer bereaved parents a way to memorialize children. | Rebuild confidence, self-esteem, and independence through personalized activities. | Focus on emotional and psychosocial well-being for children with serious illnesses and their families. | Legacy building focuses on expression and coping through digital storytelling; Digital storytelling (Ireland) focuses on having fun and confidence-building during the activities. | Understanding the theoretical foundation helps in maintaining the integrity of the intervention during adaptation. |
| What (Materials) | Describe any physical or informational materials used in the intervention. | Digital storytelling tools (video recordings, photographs, music), web-based platform. | Arts and crafts supplies, construction materials, science kits, sports equipment, digital devices. Plus digital storytelling tools. | Both use diverse materials tailored to their activities. | The original intervention emphasizes digital tools for storytelling; Digital storytelling (Ireland) intervention uses a broader range of physical materials. | Ensure materials are culturally relevant and easily accessible in the new context. |
| What (Procedures) | Describe each of the procedures, activities, and/or processes used in the intervention. | Videographer records child’s responses, incorporates chosen media into a digital story via web-based platform. | Story and activities selected based on family’s energy levels and interests (e.g., dance, construction, science). | Structured activities designed to achieve emotional and psychological outcomes. | Digital storytelling (USA) involves creating a digital narrative; Digital storytelling (Ireland) involves digital storytelling and other activities based on participant interests. | Procedures should be adaptable to meet individual family needs while maintaining core elements. |
| Who Provided | For each category of intervention provider, describe their expertise, background, and any specific training given. | Developed by a research team with expertise in nursing, bereavement, pediatric palliative care; involves videographers. | Delivered by the Barretstown Outreach Team, trained professionals in therapeutic and recreational settings. | Delivered by specialized teams trained for their target populations. | The USA intervention team includes university-trained videographers; The Ireland intervention team has professionals in therapeutic recreation. | Continuous training and support for staff are crucial for maintaining intervention quality and addressing emotional demands. |
| How | Describe the modes of delivery (e.g., face-to-face or by some other mechanism such as internet or telephone) of the intervention and whether it was provided individually or in a group. | In-person for recording sessions or web-based for story creation, delivered individually or in a family setting. | In-person at Barretstown or family’s home, delivered through structured sessions. | Flexible delivery modes to accommodate children and families. | Digital storytelling (USA) can be online or in-person; Digital storytelling (Ireland) is primarily face-to-face. | Flexibility in delivery methods can enhance accessibility and participation. |
| Where | Describe the type(s) of location(s) where the intervention occurred, including any necessary infrastructure or relevant features. | Varied settings including hospitals, homes, or fully online. | Barretstown or participant’s home, ensuring a comfortable and familiar setting. | Implemented in home settings, providing a familiar environment. | Digital storytelling (USA) offers more flexibility in location; Digital storytelling (Ireland) is more location-specific but expanding to home settings. | Adapt the setting to be comfortable and accessible, ensuring it supports the intervention goals. |
| When | Describe the number of times the intervention was delivered and over what period of time, including the number of sessions, their schedule, and their duration, intensity, or dose. | Single session with flexible completion time, final stories averaged 7 minutes 40 seconds. | Structured 2-hour sessions with introduction, activities, and wrap-up. | Both are structured with defined beginning and end, designed to be completed in a specific timeframe. | Digital storytelling (USA) has a flexible single-session approach; Digital storytelling (Ireland) has a structured, time-bound session format. | Flexibility in timing can cater to different family schedules and needs. |
| Tailoring | If the intervention was planned to be personalized, titrated, or adapted, then describe what, why, when, and how. | Highly personalized, tailored to each child’s preferences for music, photographs, and content. | Challenges and activities tailored to individual abilities and preferences. | Highly personalized to meet the specific needs and abilities of each participant and family. | Digital storytelling (USA) personalization through storytelling elements; Digital storytelling (Ireland) adjusts activities and stories based on family input. | Tailoring interventions to individual needs enhances relevance and impact. |
| Modifications | If the intervention was modified during the study, describe the changes (what, why, when, and how). | Transition from in-person to web-based to reflect technological advancements and remote accessibility needs. | Activities adjusted based on real-time observation and camper feedback. | Flexibility and adaptability in response to participant feedback and evolving needs. | Legacy building intervention adapted to include online platforms; Digital storytelling (Ireland) adjusts based on real-time feedback. | Modifications should be documented and assessed for their impact on outcomes. |
| How Well (Planned) | If intervention adherence or fidelity was assessed, describe how and by whom, and if any strategies were used to maintain or improve fidelity, describe them. | Qualitative feedback from participants, quantitative measures of quality of life and communication improvements. | Measured by camper engagement, personal challenges achieved, positive feedback from families. | Mechanisms to assess impact focus on participant engagement, achievement, and feedback. | Legacy building uses qualitative and quantitative measures; adapted intervention  measures engagement and achievements. | Regular monitoring and feedback help maintain fidelity and improve the intervention. |
| How Well (Actual) | If intervention adherence or fidelity was assessed, describe the extent to which the intervention was delivered as planned. | Feasibility, acceptability, potential benefits noted, though outcomes varied by context and individual. | Barretstown camps activities success is measured by positive feedback and engagement at Barretstown camps. | Both have mechanisms to ensure intervention fidelity. | Legacy building has varied outcomes by context; Barretstown activities that added to digital storytelling, consistently positive feedback and engagement. | Actual delivery should be evaluated to identify and address any deviations from the planned intervention. |
