# Supplementary material for: Digital storytelling as a memory-making intervention for children and families in paediatric palliative care in Ireland: an adaptation study
Source: Front Public Health. 2026 Jan 13;13:1690798. doi: 10.3389/fpubh.2025.1690798 (PMC12835261; doi:10.3389/fpubh.2025.1690798)
Supplement: Supplementary file 2 [file Table_2.docx]

**An Overview of Adapted Digital Storytelling Memory-Making Intervention Manual**

**Table of Contents**

1. Introduction
2. Background
3. Intervention Overview
4. Getting Started
5. Implementation
6. Barretstown Integration
7. Guiding Storytelling Questions
8. Training and Requirements

**1. Introduction**

This manual provides guidelines for implementing a Digital Storytelling memory-making intervention for children with life-limiting and life-threatening conditions and their families. Adapted from a U.S.-based model, this intervention has been tailored for the Irish context through a collaborative process that integrated expertise from the original intervention, relevant Irish stakeholders, and insights from parents with prior memory-making experiences.

The goal is to create meaningful digital stories that serve as treasured keepsakes, enhance family bonding, and support emotional expression and coping. The intervention is designed as a family-centred activity, weaving memory-making into everyday routines to reduce anxiety. By presenting the activity as routine family storytelling rather than focusing on palliative care, we aim to create a comfortable and inclusive environment for children and their families.

This manual will guide delivery during the pilot phase, with feedback from facilitators and families used to refine the materials before broader implementation.

**2. Background**

The Digital Storytelling Memory-Making Intervention is grounded in the principles of Dignity Therapy, digital storytelling, and legacy-building. This framework integrates theoretical foundations, evidence-based practices, and practical steps to ensure the intervention supports children with life limiting conditions and their families.

**Dignity Therapy in Paediatric Palliative Care**

Dignity Therapy is a psychotherapeutic intervention designed to help terminally ill patients reaffirm their sense of dignity and meaning. Developed by Dr. Harvey Chochinov, it involves structured life review interviews that result in a legacy document.

**Key Components of Dignity Therapy (Chochinov et al.):**

- **Generativity:** Leaving something meaningful for future generations.
- **Continuity of Self:** Articulating how one wishes to be remembered.
- **Role Preservation:** Highlighting roles in family and community.
- **Maintenance of Pride:** Reflecting on achievements and contributions.
- **Hopefulness:** Expressing hope despite the circumstances.
- **Aftermath Concerns:** Addressing concerns about loved ones’ welfare after the patient’s passing.
- **Care Tenor:** Ensuring quality and compassionate care.

**Digital Storytelling as a Legacy Intervention**

Digital storytelling combines therapeutic storytelling with digital media, allowing children to create narratives that capture their experiences, memories, and messages for loved ones. Research by Akard et al. has shown the feasibility and effectiveness of this approach in paediatric palliative care.

**The Process of Digital Storytelling (Akard et al):**

- **Interviewing:** Guiding children through questions about their lives, interests, and relationships.
- **Media Collection:** Gathering personally significant photographs, videos, and music.
- **Story Creation:** Combining these elements to create a digital story.
- **Review and Sharing:** Finalising and sharing the story with the child’s family.

**Conceptual Framework**

This framework examines the relationships between legacy interventions, family coping, and expected outcomes.

**Inputs:** Staff, training materials, digital tools, and the participation of families, interventionists, researchers, and policymakers.

**Activities:** Digital storytelling sessions and facilitator training.

**Anticipated Outputs:** Number of digital stories created, training sessions completed, and families engaged.

**Anticipated Outcomes:** Short-term (improved family communication), Intermediate (enhanced family bonding and coping), and Long-term (improved bereavement outcomes and quality of life).

**3. Intervention Overview**

The intervention is a culturally-adapted version of Akard’s model, developed based on a systematic literature review and extensive stakeholder consultations. These consultations emphasized the need for flexibility, cultural sensitivity, and accessibility to accommodate diverse communication abilities and family structures.

The intervention actively involves the entire family, allowing children to decide who participates. Activities are personalised to fit each child’s abilities, ensuring that even non-verbal children can engage meaningfully. Memory-making is woven naturally into family routines to avoid feeling burdensome.

Support is provided at all stages, both to families and trained staff. The program’s logistics cover practical needs like travel and flexible settings (e.g., home, virtual, or hospice) and resources for staff training and equipment. Families are supported in revisiting digital memories, with consistent communication and follow-up offered. Privacy protocols are strictly maintained, ensuring family stories are shared securely within healthcare frameworks.

**4. Getting Started**

**Suitable Participants:**

- **Children:** Aged 5 to 12 years, diagnosed with life-limiting or life-threatening conditions. The program is designed to be inclusive of diverse communication abilities.
- **Family Members:** Those closely involved in caregiving, including siblings and extended family, are encouraged to participate to foster collaboration and bonding.

**A Flexible and Personalized Approach**

The program encourages the child to select questions and methods that help them tell their story. The final product is a 10–15-minute video, co-created by the child and family, featuring their story with chosen photos and music.

**Preparation Materials**

Families will receive preparation materials, including guiding questions, examples of activities and a sample storytelling video. This helps families visualise the process and understand how to participate actively.

### **Steps for Delivering the Intervention**

#### **1. Preparation Phase (Pre-Visit)**

**Step 1: Planning and Ethical Considerations**

- **Develop Materials:** Create and distribute brochures or guides introducing the intervention to families, and stakeholders.
  - Include GDPR and ethical considerations (e.g., storage of digital media and informed consent).
- **Purpose:** Ensure clarity about the program goals and expectations for families.

**Step 2: Initial Family Contact**

- **Expression of Interest:** Families express interest in participating, possibly referred by medical or other teams.
- **Initial Call (Phone/Zoom):**
  - Introduce the intervention as a fun, family-centered storytelling activity, avoiding clinical or sensitive medical terms.
  - Outline the process, session length, and final product.
  - **Checklist Review:** Establish which program fits the child’s needs and outline preparation steps (materials, timeline, etc.).

#### **2. Preparation Phase (Before the Intervention)**

**Step 3: Provide Materials to Families**

- Distribute preparation materials:
  - **Guiding Questions**: Include simplified and adaptable questions for storytelling.
  - **Checklist:** Suggestions for gathering photos, videos, music, or other meaningful items (consider cultural and family-specific elements).
  - Other forms like Consent and Assent forms for sign from Researchers to review and sign.

**Step 4: Follow-Up and Personalization**

- Conduct follow-up calls or virtual meetings with families to:
  - Confirm they’ve received and understood the materials.
  - Discuss family preferences, such as:
    - Communication styles (e.g., verbal/non-verbal).
    - Emotional energy levels and mobility needs.
    - Family dynamics and inclusion of siblings or extended family.
  - Confirm communication methods, preferred participants, location, and timing.
  - Use the Guiding Storytelling Questions to gather key information on interests, sensitivities, and practical needs for storytelling.
  - Help families start gathering media and brainstorming ideas for their digital story.
  - Conduct follow-up calls with families a few days before the intervention day.

#### **3. Delivery Phase**

**Step 5: Day of the Intervention**

- **Facilitating Storytelling Sessions:**
  - Staff or facilitators support families during storytelling sessions (1-2 hours max).
  - Flexible options include:
    - Guiding children through answering selected questions.
    - Using non-verbal methods (e.g., photo selection or sensory activities) if needed.
    - Recording family interactions or creative activities like music or drawing.
  - Keep the tone light and routine to avoid anxiety.
- **Tech Setup:** Ensure all technology (e.g., cameras, laptops) is ready for smooth facilitation.
- **Family Interaction:** Encourage bonding by involving the whole family in the process.

#### **4. Post-Intervention Phase**

**Step 6: Gathering the delivery team feedback**

- Gather detailed feedback from families and staff to assess the program’s effectiveness and identify areas for improvement.

**Step 7: Creating the Digital Story**

- Compile the gathered media (photos, videos, music) and storytelling recordings into a cohesive digital story (10–15 minutes).
- Involve families' ideas about how they want this final product looks on the intervention day and make sure in the editing process to ensure their voices and preferences are honored.
  - Verify that all media shared respects cultural and personal boundaries.

**Step 8: Feedback and Closure**

- Share the completed digital story with families (ensure proper consent and privacy protocols).
- **Reflection and Follow-Up:** Conduct a debriefing session:
  - Allow families to provide feedback on their experience (2-3 weeks after video delivered).
  - Offer emotional support for families and facilitators.
- Use surveys, interviews, or informal discussions to explore:
  - Families’ experiences and satisfaction with the storytelling process.
  - Suggestions for enhancing support or refining the storytelling framework.
- Incorporate this feedback into future program iterations to ensure continued relevance and adaptability to participants’ emotional, cultural, and logistical needs.

**5. Implementation**

The storytelling intervention is designed to be flexible and sensitive to the unique needs of each family.

- **Facilitation:** An activity leader facilitates the storytelling intervention, centering on the child–family unit.
- **Family Involvement:** Both children and their families are encouraged to participate in interactive activities, including conversations and creative tasks during storytelling sessions. These sessions are audio/video-recorded based on participants' preferences.
- **Session Duration:** Each session lasts up to 2 hours; however, the timing and length are flexible, depending on the child’s physical condition and the depth of the family’s narrative sharing.
- **Documentation and Final Product:**
  - Recorded sessions are transcribed and edited into a cohesive video narrative.
  - Narrations from children and families combined into a single document or kept separate, depending on the participants’ preferences.
  - The final product includes the video narrative as its core, supplemented with cherished items such as crafts or photos provided by participants.
- **Keepsake Delivery:** The generativity entity is returned to children and families as a treasured memento.

**Intervention Setting:** The location of the intervention should be determined based on the preferences of children and families. This may include the home, private meeting rooms, or other comforting spaces, with their favourite items such as toys or flowers present.

**Implementation Guidelines:**

- **About Self and Connectedness with Others**: Questions should balance verbal and non-verbal expressions. Creative activities (drawing, selecting images, or recording sounds) can be used for non-verbal children to capture emotions and memories.
- **Cultural and Age-Specific Adaptations:** Tailor the questions to age, cognitive ability, and cultural background. For younger children or those with developmental challenges, simplify questions and support alternative methods for sharing their stories.

**Integration of Stakeholder Feedback:**

- **Family Engagement:** Families are encouraged to join in the memory-making process. Providing mock-ups and examples helps families better understand how to participate actively.
- **Support for Staff and Families**: Ensure families are supported through ongoing interactions before, during, and after the intervention. Consistent staff support is crucial for managing emotional and logistical aspects.

**6. Barretstown Integration**

The intervention incorporates Barretstown’s therapeutic recreation model, ensuring it integrates into existing support structures for families. Activities such as sensory play and art-making are offered as alternative ways for non-verbal children or those with different communication abilities to engage.

Examples of activities that can be integrated include:

**High Energy:**

- Barretstown Dance Workshop
- Crazy Construction
- Wacky Science

**Medium Energy:**

- Movie Making (With digital storytelling)
- Tie-dye
- Drama Workshop

**Low Energy:**

- Arts and Crafts
- Giant Bubbles
- Sensory Play

**7.Guiding Legacy Questions (prompts)**

The following questions can be adapted for younger or non-verbal participants by focusing on more concrete memories or non-verbal methods of expression, such as selecting favorite images or objects.

**Section: About Self**

1. What is your favourite colour? Food? Sports team or athlete? Hobbies? TV show or movie? Music? Song? Places to go?
2. What is your most special personal belonging and why?
   **OR**: Do you have a favourite toy, book, or object? Why is it special to you?
3. Thinking way back to a long time ago, what is your favourite memory?
   **OR**: Can you think of a happy memory? What do you remember about it?
4. What is the funniest thing that you’ve ever done?
   **OR**: What is something that made you laugh a lot?
5. Pretend you wrote a story about yourself. What is the story about? What part of the story would you want others to remember the most?
6. What are the most important things you have done, and what do you feel most proud of? What is something you are excited about doing soon?
7. Please share what you have done that makes you most happy?
8. Is there anything else you’d like to share about yourself?

**Section: Connectedness with Others**

1. Who are the people who are important to you (e.g., family, friends)? Can you tell me something special about them?
2. Who is your hero and why? How would you describe him/her?
   **OR:** Do you have someone you look up to? What do you like most about them?
3. Is there anything special you’d like to say to the people you care about?

### **8. Training and Requirements**

The training program prepares the Barretstown's team with the skills, sensitivity, and resilience required to implement the intervention effectively.

#### **Emotional Sensitivity and Implementation:** focuses on empathy, effective communication, and cultural sensitivity

#### **Family-Centered Personalization:** Prioritizes tailoring the intervention to the unique needs of each family, including non-verbal communication and flexibility.

#### **Palliative Care Fundamentals:** Provides a foundational understanding of paediatric palliative care principles.

#### **Digital Storytelling and Technical Skills:** Trains staff to guide families in creating meaningful digital stories and managing the technical aspects.

#### **Role-Playing and Feedback:** Uses hands-on training and simulated scenarios to build confidence.

#### **Staff Well-Being and Support:** Emphasizes self-care and provides access to support networks to help staff manage the emotional demands of the role.

**References:**

1. Chochinov, H. M., Hack, T., McClement, S., Kristjanson, L., & Harlos, M. (2002). Dignity in the terminally ill: a developing empirical model. *Social science & medicine (1982), 54*(3), 433–443. <https://doi.org/10.1016/s0277-9536(01)00084-3>
2. Chochinov, H. M., & McKeen, N. A. (2011). Dignity therapy. Handbook of psychotherapy in cancer care, 79-88.
3. Akard, T. F., Gilmer, M. J., Friedman, D. L., Given, B., Hendricks-Ferguson, V. L., & Hinds, P. S. (2013). From qualitative work to intervention development in pediatric oncology palliative care research. *Journal of pediatric oncology nursing : official journal of the Association of Pediatric Oncology Nurses, 30*(3), 153–160. <https://doi.org/10.1177/1043454213487434>
4. Akard, T. F., Dietrich, M. S., Friedman, D. L., Hinds, P. S., Given, B., Wray, S., & Gilmer, M. J. (2015). Digital storytelling: an innovative legacy-making intervention for children with cancer. *Pediatric blood & cancer*, 62(4), 658–665. <https://doi.org/10.1002/pbc.25337>
